# Targeting tumor-associated macrophages for the immunotherapy of glioblastoma: Navigating the clinical and translational landscape

**DOI:** 10.3389/fimmu.2022.1024921

**Published:** 2022-10-13

**Authors:** Zide Wang, Hanlin Zhong, Xiaohong Liang, Shilei Ni

**Affiliations:** ^1^ Department of Neurosurgery, Qilu Hospital of Shandong University, Cheeloo College of Medicine and Institute of Brain and Brain-Inspired Science, Shandong University, Jinan, China; ^2^ Jinan Microecological Biomedicine Shandong Laboratory and Shandong Key Laboratory of Brain Function Remodeling, Jinan, China; ^3^ Key Laboratory for Experimental Teratology of Ministry of Education, Key Laboratory of Infection and Immunity of Shandong Province and Department of Immunology, School of Basic Medical Sciences, Cheeloo Medical College of Shandong University, Jinan, China

**Keywords:** glioblastoma, tumor-associated macrophage, immunotherapy, tumor microenvironment, immunosuppression

## Abstract

Tumor-associated macrophages (TAMs) can directly clear tumor cells and enhance the phagocytic ability of immune cells. An abundance of TAMs at the site of the glioblastoma tumor indicates that TAM-targeting immunotherapy could represent a potential form of treatment for this aggressive cancer. Herein, we discuss: i) the dynamic role of TAMs in glioblastoma; ii) describe the formation of the immunosuppressive tumor microenvironment; iii) summarize the latest clinical trial data that reveal how TAM function can be regulated in favor tumor eradication; and lastly, iv) evaluate the implications of existing and novel translational approaches for treating glioblastoma in clinical practice.

## 1 Introduction

Glioblastoma (GBM) is the most common malignant tumor affecting the central nervous system (CNS), which in 2021, was classified as a grade 4 tumor by the World Health Organization (WHO) (1). The median survival of patients with GBM undergoing radiotherapy plus temozolomide treatment is ~14.6 months, with 89% of patients dying after 5 years (2, 3). Thus, a new therapeutic approach is urgently needed to overcome the limitations of the currently available treatment options, such as the low concentration of the tumor-targeting drug at the tumor site and the strong adverse effects associated with some forms of treatment. Immunotherapies like immune checkpoint blockade (ICB) significantly extended patient overall survival (4). The discovery of functional lymphatic vessels dispelled the traditional view of the brain as an immune privileged organ. In addition, evidence has emerged showing that immune cells migrating from the draining deep cervical lymph nodes could directly prevent GBM growth and metastasis (5–9).

The immune cell composition of the GBM tumor is as follows: 20–40% leukocytes, up to 80% tumor-associated macrophages (TAMs), and much fewer tumor-infiltrating lymphocytes (TILs), whose activity is limited due to the unique immunosuppressive tumor microenvironment (TME) (10–13). Thus, a potential therapeutic strategy for GBM would be to target TAMs.

Herein, we discuss recent advances in the interactive relationship between GBM and TAMs and summarize the significant breakthroughs in the immunotherapeutic approaches that focus on targeting this cell subset in GBM while overcoming some limitations of conventional immunotherapies. A comprehensive understanding of the interaction between TAM-targeting clinical and translational research will further promote the development of therapeutic strategies in GBM.

## 2 The role of TAMs in GBM

**Figure 1:**
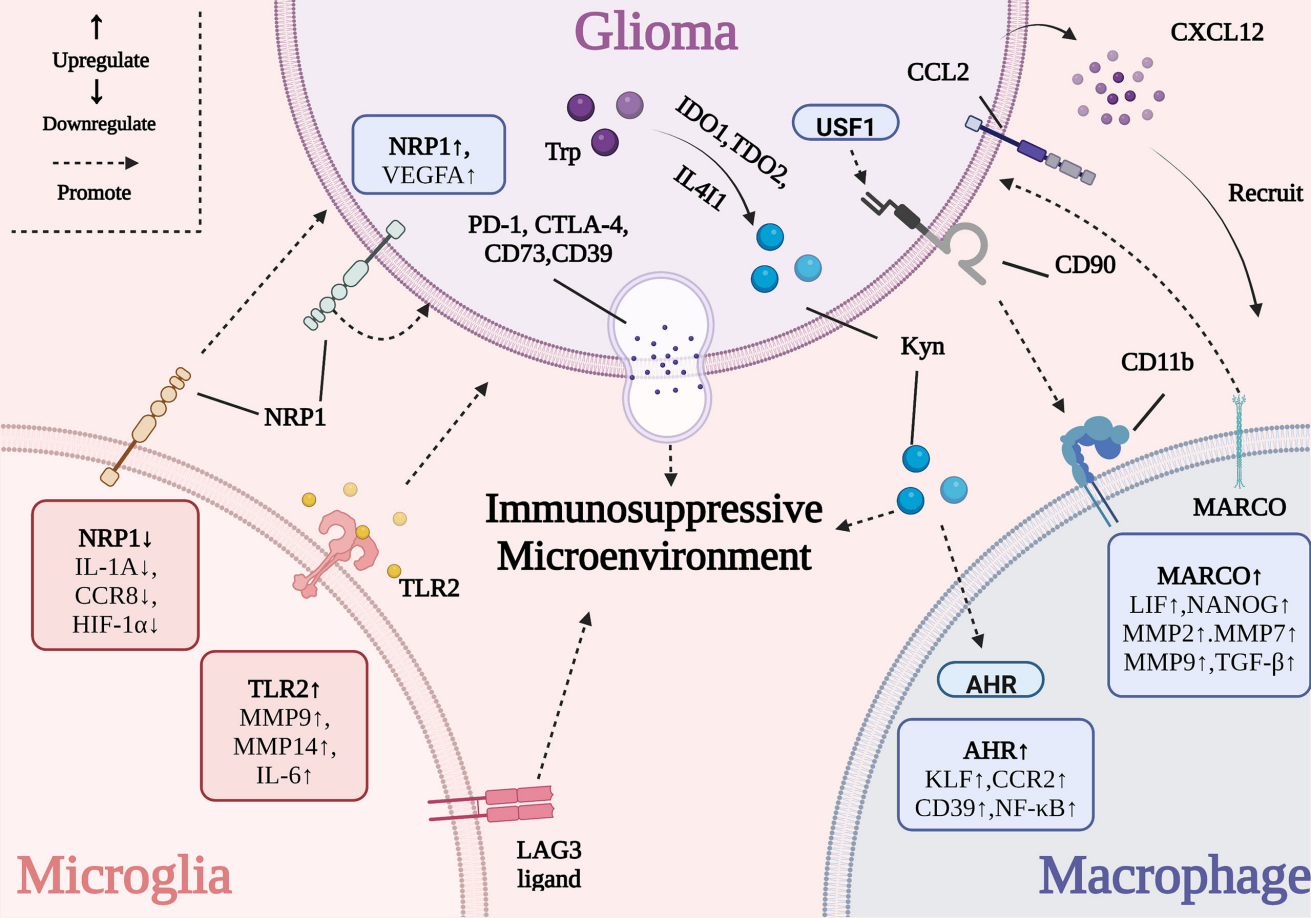
The interactive mechanism between GBM and TAMs. The activation of tumor-promoting genes (e.g., *Tlr2*, *Marco*, and *Ahr*) in TAMs influences tumor growth, metastasis, angiogenesis and immune evasion. The overexpression of some genes in GBM (e.g., *Ido1*, *Tdo2*, *Il4i1*, and *Usf1*) damages the activity of TAMs.

The TAMs residing with the tumor tissue consist of bone marrow-derived macrophages (BMDMs) and tissue-resident macrophages (TRMs) (14). And the TAMs contribute to tumor proliferation (13, 15), invasion (16), metastasis (17), and angiogenesis (18, 19) in various cancer types. However, the TRMs in different organs might play contradictory roles in tumor progression. For instance, the TRMs are demonstrated to promote lung tumor invasiveness and metastasis while the ablation of TRMs in breast cancer has no impact on tumor growth (20–22). Therefore, how the unique TRMs microglia (MG) affect the GBM progression merit further investigation. Moreover, the distribution of TAMs in GBM is spatially heterogeneous: BMDMs are recruited to the hypoxic tumor parenchyma, while MG are localized to the peritumoral regions (23, 24). Herein, we investigated the relationship between TAMs and GBM outlined in **Figure 1**.

### 2.1 The polarization of TAMs in GBM

**Figure 2:**
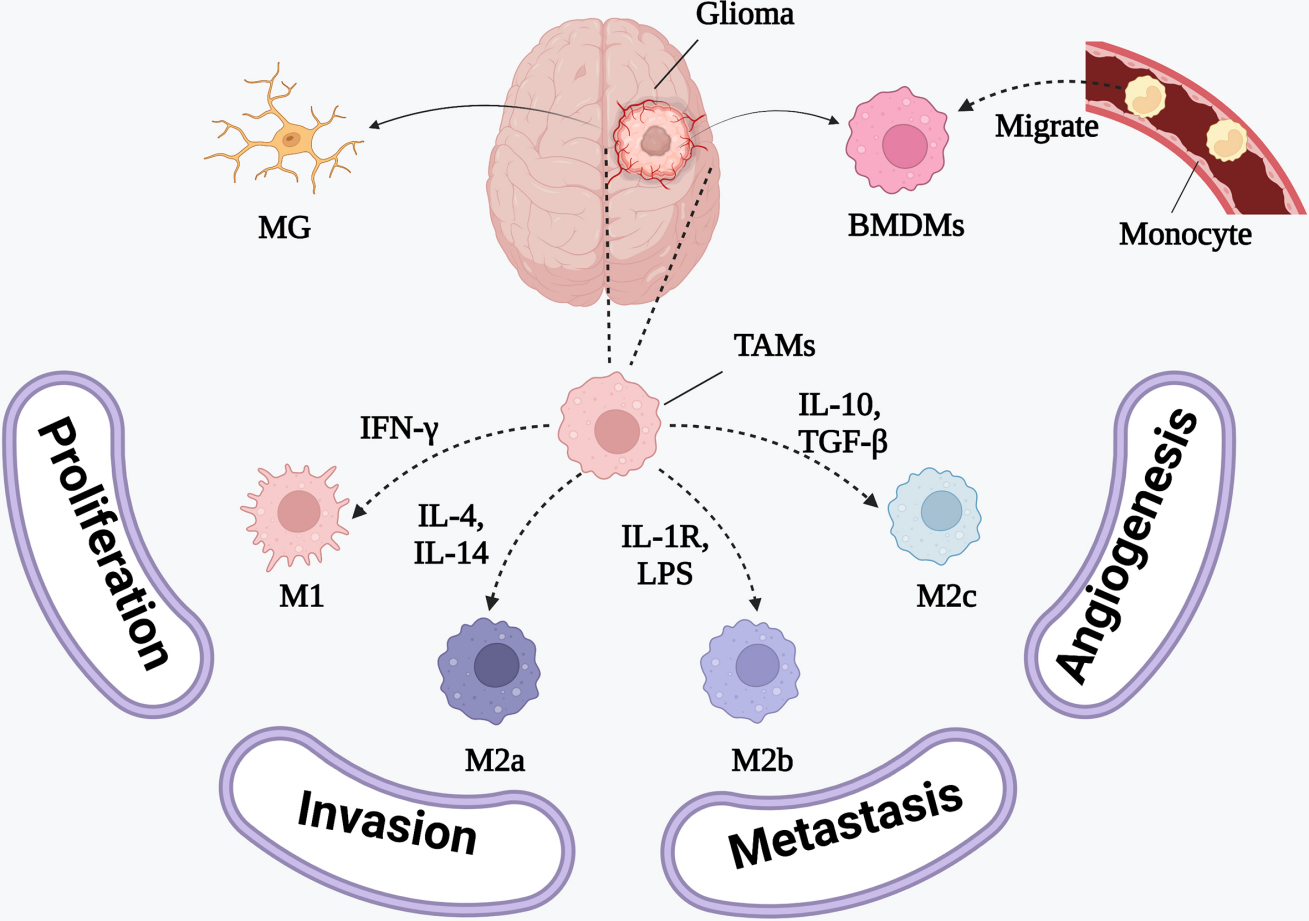
The phenotypes of BMDMs and the inducing cytokines. M1 polarized macrophages are induced by IFN-γ to release proinflammatory cytokines. M2 polarized macrophages releasing anti-inflammatory cytokines consist of IL-4 and IL-13 activated M2a polarized macrophages, IL-1R ligands or immune complexes plus LPS activated M2b polarized macrophages and TGF-β and IL-10 activated M2c polarized macrophages.

TAMs are usually simply divided into interferon-gamma (IFN-γ)-activated M1 polarized macrophages and IL-4-activated M2 polarized macrophages (**Figure 2**). M1 macrophages are capable of releasing proinflammatory cytokines, presenting antigens to immune cells, and phagocytosing GBM cells. Meanwhile, M2 macrophages contribute to the immunosuppressive TME and promote tumor progression and invasion (25). M2 macrophages are the predominant macrophage subset at the GBM tumor site. However, in GBM, they often exist as a heterogeneous population comprising the IL-4- and IL-13-activated M2a subset, the M2b subset (activated by IL-1R ligands or immune complexes plus lipopolysaccharides, LPS), and the M2c subset (activated by IL-10 and transforming growth factor beta, TGF-β) (26, 27). However, tumor samples from patients with GBM have revealed that TAMs are actually a mixed M1/M2 subset expressing both M1 and M2 surface markers, which might be related to GBM heterogeneity (28, 29). Thus, future studies should initially focus on accurately classifying TAMs before investigating their role in the GBM microenvironment.

### 2.2 The interaction between MG and GBM

MG originate from the yolk sac of primitive myeloid progenitors and can promote the development of the brain and protect neuronal function. The colony-stimulating factor-1 (CSF-1) maintains MG self-renewal *in situ* (30, 31). Studies in mice have demonstrated that MG can be further divided into at least two subsets: i) homeostatic MG, and ii) neurodegenerative MG, which are induced by the triggering receptor expressed on myeloid cells 2 (TREM2) - apolipoprotein E (APOE) pathway; both of these MG subsets play a key role in detrimental neurodegenerative disorders such as Alzheimer’s disease (32, 33). However, current studies have mainly focused on the whole MG population and ignored the specialized functions of specific MG subsets. Therefore, the roles of different types of MG in GBM still need to be explored.

The overexpression of the neuropilin-1 (*Nrp1* in mice and *NRP1* in humans) in GBM has been shown to support tumor cell growth, metastasis, and immune evasion (34). *Nrp1* promotes angiogenesis *via* the release of vascular endothelial growth factor A (VEGFA), which causes TAMs to adopt an anti-inflammatory phenotype by decreasing the secretion of pro-inflammatory factors and contributing to the immunosuppressive TME (34). *Nrp1*-deficient MG promoted a reduction in the levels of interleukin (IL)-1α, CC motif chemokine receptor 8 (CCR8), and hypoxia-inducible factor 1-α (HIF1-α) and therefore delayed the progress of angiogenesis while promoting cytotoxic T cell and MG infiltration and decreasing the proportion of BMDMs in the tumor tissue. *Nrp1*-MG-knockout (*Nrp1* ^MGKO^) mice had smaller tumors and longer overall survival, compared to wild-type animals (35). Besides, high *Nrp1* expression showed an inverse correlation with the prognosis of GBM patients (36). Therefore, *Nrp1* could represent a potential biomarker for predicting the prognosis of patients with GBM and serve as a therapeutic target in combination with currently available standard treatment forms, after conducting large-scale clinical experiments.

Additionally, Toll-like receptor 2 (TLR2) was correlated with GBM malignancy. It was observed that the *Tlr2* expression level in the MG of mice was markedly higher in high-grade GBMs than in low-grade GBM tumors (37). The activation of TLR2-induced matrix metalloprotease (*Mt1-mmp)* and *Mmp9* expression in MG and resulted in tumor expansion and metastasis. Meanwhile other TLR subtype-specific agonists such as polyinosinic: polycytidylic acid (poly I:C; a TLR3 agonist), flagellin (a TLR5 agonist), and polyuridine (polyU, a TLR7/8 agonist) had little effect on *Mt1-mmp* gene expression (38, 39). Recently, ortho-vanillin (O-Vanillin) was shown to block TLR2-mediated signaling and lower the levels of IL-6, inducible nitric oxide synthase (iNOS), MT1-MMP, and MMP9, to attenuate GBM growth and invasion (40). In contrast, Panagioti et al. tried to reverse the local immunosuppressive TME in GBM by promoting TLR2 activation. This was achieved using the *Helicobacter pylori*-derived neutrophil-activating protein (NAP), which enhanced the outcome of anti-programmed cell death 1 (PD-1) immune-checkpoint blockade by activating TLR2 signaling (41). These seemingly contradictory results suggest that future immunotherapy designs should take into consideration the combined effects of TLR2 activation or inhibition rather than focus on isolated signaling pathways.

The high expression of major histocompatibility complex class II (MHC-II; the canonical lymphocyte-activation gene 3 ligand, LAG3 ligand) on MG can elevate the expression of exhaustion-associated biomarkers (e.g., LAG3) in T cell populations, contributing to the formation of the immunosuppressive TME (42). Meanwhile, a close correlation between the tumor suppressor protein p53 (TP53) and MG infiltration was identified. Mutant TP53^R248L^-overexpressing GBM cells significantly increased the expression of C-C motif chemokine ligand 2 (CCL2). This led to the recruitment of BMDMs and a reduction in the proportion of MG, which worsened the prognosis of patients with GBM (43). To date, there are few studies investigating the effect of GBM on local MG, and these complex interactions need to be further explored.

### 2.3 The interaction between BMDMs and GBM

Unlike tissue-resident MG, BMDMs are mainly derived from monocytes in the blood (24). GBM cells disrupt the integrity of the blood-brain barrier (BBB) during tumor initiation and release multiple cytokines such as the chemokine stromal cell-derived factor 1-α (CXCL12) to recruit monocytes or macrophages to the tumor site (44, 45). BMDMs are preferentially localized to the perivascular spaces within the tumor parenchyma due to their ability to survive in a hypoxic environment. Thus, the spatial heterogeneity of TAMs in GBM is unique (23, 24).

The presence of BMDMs can promote the growth and proliferation of GBM. For instance, TAMs exhibiting a high expression of the macrophage receptor with collagenous structure (MARCO) maintain the stemness of GBM stem cells (GSCs) by upregulating a set of stemness-associated factors such as leukemia inhibitory factor (LIF) and the homeobox protein NANOG. This in turn promotes tumor proliferation *via* the secretion of TGF-β and the metastasis of GBM through the augmented expression of *Mmp2*, *Mmp7*, and *Mmp9* (46, 47). In addition, the mixed culture system consisting of GBM cells expressing high levels of upstream stimulating factor 1 (USF1) and human macrophages enhanced the stemness of GSCs (48). CD90, a glycosylphosphatidylinositol-anchored protein regulated by USF1, has been shown to interact with macrophage surface integrins such as CD11b and promote the adhesion of macrophages and tumor cells, thus accelerating the interaction between GBM and BMDMs (48). At present, the mechanism underlying the role of BMDMs in GBM is unclear and requires delineation to provide a sufficient theoretical basis for the development of TAM-targeting therapies in GBM.

GBM cells have a profound and lasting effect on macrophage function by shaping the immunosuppressive TME. GBM cells deliver immunosuppressive proteins such as CD73, CD39, programmed cell death ligand 1 (PD-L1), and cytotoxic T lymphocyte-associated protein 4 (CTLA-4) into the local microenvironment through extracellular vesicles, leading to immune cell function impairment and tumor progression (49). The expression of IL-4-induced-1 (IL4I1), tryptophan 2,3-dioxygenase 2 (TDO2), and indoleamine 2,3-dioxygenase 1 (IDO1) in GBM initiates the catabolism of tryptophan (Trp) into kynurenine (Kyn) to activate aryl hydrocarbon receptor (AHR) in TAMs, inducing the upregulation of CCR2 and Krüppel-like factor 4 (KLF4) (50, 51). Furthermore, the activation of AHR also suppresses nuclear factor-κB (NF-κB) signaling, which recruits M2 macrophages into the tumor parenchyma and causes immune suppression. Meanwhile, the AHR-mediated upregulation of CD39 has been demonstrated to simultaneously cooperate with CD73 in the generation of adenosine monophosphate (AMP) from ATP and produce nucleotide adenosine, causing the suppression of CD8^+^ T cell immunity and the immune evasion of GBM (50–52). CD73 is regarded as a novel target for GBM immunotherapy and blocking CD73 had been shown to prolong the survival of patients with GBM receiving anti-PD-1 or anti-CTLA-4 treatment (53). However, Abdelfattah et al. performed a single-cell analysis of human GBM to highlight the absence of CD73 from GBM-associated myeloid cells (54). Therefore, the responsiveness of patients to such targeted therapies should be used as a key criterion for determining the immune therapeutic targets and strategies in GBM.

BMDMs and GBM cells collectively contribute to the immunosuppressive TME of GBM. The abundance of signal transducer and activator of transcription 3 (STAT3) in GBM has been demonstrated to promote *AHR* expression and cooperate with HIF-1α to increase CD40 levels, which in turn promoted GBM immune evasion and STAT4-mediated PD-L1 upregulation (42, 51, 55). Moreover, multiple factors have been implicated in the recruitment and polarization of BMDMs, including slit guidance ligand 2 (SLIT2) (56), Notch1 (57), PTEN (58), NF1 (59), TGF-β (47, 60, 61), M-CSF (47, 60, 61), PD-L2 (62), IL-33 (63), arginase 1 (ARG1) (64), CD47 (65), and CD70 (66). The complex network of interactions that exist between BMDMs and GBM cells within the unique TME needs to be further explored, so as to provide robust guidelines for effective GBM treatment.

## 3 TAM-targeting therapies for GBM

The current standard GBM strategy is surgical resection combined with oral temozolomide (TMZ) and radiotherapy (2, 3). Moreover, many novel approaches recently have emerged in GBM therapy, such as 5-aminolevulinic acid, bevacizumab, and tumor treating fields (67–69). However, the therapeutic efficacy of these is still limited. Thus, TAM-targeting immunotherapy is deemed as a potential method to deal with the problems in GBM treatment.

### 3.1 TAM recruitment prevention

As previously alluded to, M2 TAMs are implicated in GBM progression and invasion. Consequently, preventing TAM recruitment to the tumor site might be a potential strategy for eliminating the GBM tumor. Ongoing clinical trials are concentrating on achieving this with inhibitors targeting the colony-stimulating factor 1 receptor (CSF1R), angiopoietin-2 (ANG2), and CXCR4 (**Table 1**).

**Table 1:** The TAM-targeted clinical trials in GBM.

Agent	Combination partners	Trial phase	Patients (n)	Reported biological responses	Clinical trials registry identifier
**CSF1R Inhibitors**					
Pexidartinib	–	II	38	Monocyte↓	NCT01349036
**ANG2 Inhibitor**					
MEDI3617	Bevacizumab	I	13	ORR:0%	NCT01248949
Trebananib	Bevacizumab	II	130	NR	NCT01609790
**CXCR4 Inhibitors**					
Plerixafor	TMZ, Radiation	I/II	29	CXCL12↑	NCT01977677
Plerixafor	Bevacizumab	I	26	Lymphocytes↑ Monocytes↑ CXCL12 ↑ ANG2, sMET, IL-8↓	NCT01339039
**CD40 agonists**					
APX005M	–	I	45	NR	NCT03389802
2141-V11	D2C7-IT	I	30	NR	NCT04547777
**TLR agonists**					
**TLR3**					
Poly-ICLC	–	II	47	50% LGG respond 25% HGG respond	NCT01188096
	Radiation	II	31	NR	NCT00052715
	TMZ, Radiation	II	97	NR	NCT00262730
	GAA/TT-Peptide Vaccine	I	13	91% respond	NCT00795457
	Peptide Vaccines	I	10	55% respond	NCT00874861
	IMA950 Peptide Vaccine	I/II	19	NR	NCT01920191
	Dendritic Cell Vaccine	I	28	TNF-α↑ IL-6↑ Lymphocytes↑	NCT00068510
**TLR4**					
HSPPC-96	–	I	20	NR	NCT02122822
	–	II	96	NR	NCT00293423
**TLR9**					
CpG-ODN	–	II	34	No Benefit	NCT00190424
**PD-1 Inhibitors**					
Nivolumab	–	III	529	ORR:7.8%	NCT02017717
	–	II	29	CXCL10, CCL4, CCL3L1↑	NCT02550249
	Ipilimumab	I	27	No Benefit	NCT03233152
	Lirilumab	II	397	NR	NCT02813135
Cemiplimab	Veledimex	II	40	NR	NCT04006119
Pembrolizumab	Bevacizumab, Hypofractionated Stereotactic Irradiation	I	32	NR	NCT02313272
	Bevacizumab	II	80	ORR:20%	NCT02337491

ORR, objective response rate; NR, not reported; ↑, increase; ↓, decrease.

#### 3.1.1 CSF1R inhibitors

CSF1R, a type III protein tyrosine kinase receptor, is widely expressed by TAMs (70). The activation of CSF1R signaling by IL-34 or CSF1 induces the recruitment of macrophages and shifts the TAM phenotype from M1 to M2 (71–73). Pexidartinib (PLX3397), the first commercial CSF1R inhibitor approved by the Food and Drug Administration (FDA), was recently used in a phase II clinical trial of recurrent glioblastoma (NCT01349036). However, there was no significant improvement in the pexidartinib group compared to the control group even though pexidartinib was present within the GBM tumor at its therapeutic concentration. Interestingly, the progression-free survival (PFS) of two patients with mesenchymal GBM was extended as a result of pexidartinib treatment (74). However, CSF1R blockade caused the overexpression of PD-L1, inhibiting the phagocytosis and antigen-presenting capacity of macrophages (75, 76). The failure of pexidartinib in GBM does not signal the absolute failure of CSF1R inhibitors. It will be interesting to observe the outcome of a recently initiated clinical trial (currently in the recruitment phase) that will be testing the performance of another CSF1R inhibitor, BLZ-945 (NCT02829723). In addition, the mechanism of CSF1R blockade should be illustrated in different GBM cell subsets.

#### 3.1.2 ANG2 inhibitors

ANG2 is an angiogenic factor that promotes vascular destabilization and leakage by binding to the tyrosine-protein kinase receptor TIE2 (77). The hypoxic TME induces the high expression of TIE2 in TAMs, which are involved in vascular reconstruction and tumor relapse (78). Therefore, multiple ANG2 inhibitors have been applied in GBM clinical trials. Patients with recurrent malignant GBM were treated with the selective ANG2 inhibitor MEDI3617 in a phase I study (NCT01248949) (79). The results showed that both the MEDI3617 monotherapy and the combination therapy with bevacizumab were tolerated but ineffective. Moreover, the treatment regimen was associated with notable adverse events, including hypertension, proteinuria, and peripheral edema. Of these, peripheral edema was the most frequent adverse event, which led to treatment discontinuation. Similarly, another ANG2 inhibitor LY3127804 also elicited frequent adverse events like hypertension and peripheral edema (NCT02597036) (80). A phase II clinical trial using bevacizumab and trebananib (an ANG1/2 inhibitor) to treat GBM indicated that patients with recurrent GBM did not benefit from trebananib treatment (NCT01609790). On the contrary, the PFS of the control group was significantly longer than of the experimental group, which might be related to the complex interactions between ANG1 and ANG2 (81). The low response rates and numerous adverse effects are the main challenges facing the clinical use of ANG2 inhibitors. Therefore, measures to improve the efficacy and biocompatibility of ANG2 inhibitors should be put in place.

#### 3.1.3 CXCR4 inhibitors

CXCR4 is a transmembrane G-protein coupled-receptor activated by CXCL12, which is involved in tumor growth, invasion, angiogenesis, and TAM recruitment *via* the activation of signaling pathways such as MEK/ERK and PI3K/mTOR (82). Plerixafor, a CXCR4 inhibitor approved by the FDA, was combined with radiation therapy to reduce macrophage accumulation after radiotherapy (NCT01977677). The median overall survival was prolonged to 21.3 months and 93% of patients did not experience GBM progression at 6 months in the plerixafor group (83). Although, this clinical trial evaluating the use of anti‐angiogenic drugs to treat GBM achieved promising results, an increase in CXCL12 levels was observed in the patients’ plasma (84). Therefore, a phase I study (NCT01339039) using a combination of plerixafor and bevacizumab (a VEGF inhibitor) in high-grade GBM was commissioned to alleviate this side-effect of anti‐angiogenic therapy. However, this novel combination of drugs failed to produce better clinical outcomes, which might be related to the changes in the levels of cytokines such as soluble mesenchymal epithelial transition receptor (sMET), CXCL12, and IL-8 (85). Although plerixafor has shown great efficiency in some GBM clinical trials, the limited number of patients involved in these studies may obscure the possible serious adverse effects of this treatment.

### 3.2 TAM repolarization

TAMs exhibit a high level of plasticity, meaning that promoting a shift between M2 and M1 phenotypes is a potential strategy for treating GBM. CD40 and TLR agonists are currently being validated as therapeutic agents targeting TAM repolarization in GBM clinical trials.

#### 3.2.1 CD40 agonists

CD40 belongs to the tumor necrosis factor (TNF)-receptor superfamily and is primarily expressed on antigen-presenting cells. The crosslink between CD40 and its ligand CD40L has been demonstrated to assist dendritic cells (DCs) to activate T cells and reprogram TAMs to inhibit tumor growth (86, 87). Many CD40 agonists have achieved significant therapeutic efficacy in the treatment of pancreatic cancer and lymphoma (88–92). However, few clinical trials have assessed the value of using CD40 agonists to treat GBM. Two phase I clinical trials of the CD40 agonistic antibodies APX005M (NCT03389802) and 2141-V11 (NCT04547777) are currently enrolling GBM patients. The outcomes of these trials are being awaited with anticipation after a mouse study reported that the CD40 agonistic antibody induced ICB and extended the median survival of GBM model mice to 37.0 days compared with 21 days in control, indicating the tremendous potential of CD40 agonists in GBM treatment (55).

#### 3.2.2 TLR Agonists

TLRs are common pathogen recognition receptors that detect soluble factors released during cell death that are regarded as danger-associated molecular patterns (DAMPs) (93). Existing studies have linked the expression of TLR2, TLR4, and TLR9 on GBM cells to tumor proliferation, invasion, and migration (38, 94, 95). Conversely, certain TLR agonists such as poly I:C, resiquimod, and imiquimod, were used as vaccine adjuvant to suppress tumor growth *via* the reprograming and repolarizing of TAMs at the tumor site (96–99). The TLR3 agonist polyriboinosinic-polyribocytidylic acid-poly-L-lysine carboxymethylcellulose (poly-ICLC) was a common vaccine adjuvant in GBM clinical trials. For instance, therapeutic vaccination with a combination of synthetic peptide GBM-associated antigen (GAA) epitopes and poly-ICLC (NCT00795457 and NCT00874861) induced a robust IFN-γ production and prolonged the median PFS of patients with GBM to 21 months post-diagnosis. The main adverse events were slight injection site reactions and flu-like symptoms such as fever, myalgia, and fatigue (100). In addition, other clinical trials of GBM used poly-ICLC as an adjuvant to improve the efficacies of radiotherapy with TMZ (NCT00262730), a DC vaccine (NCT00068510), and a multi-peptide IMA950 vaccine (NCT01920191); with a variety of outcomes in patients (99, 101, 102). Additionally, a phase II study (NCT00190424) attempted to deliver a TLR9 agonist (oligodeoxynucleotides containing CpG motifs, CpG-ODN) to the brain to treat recurrent glioblastoma; disappointingly, this strategy was proved ineffective (103). The steps taken to select appropriate treatment combinations to maximize the anti-tumor effect while minimizing adverse events would merit further investigation.

### 3.3 Immune checkpoint blockade

In recent years, ICB has demonstrated tremendous promise as a form of cancer therapy. Several immune checkpoint inhibitors such as anti-PD-1 and anti-CTLA-4 have been used to successfully treat several types of cancer, including melanoma and non-small cell lung cancer (104). Therefore, ICB is expected to be an effective GBM treatment.

The high expression of PD-L1 on TAMs impairs their activation, proliferation, and survival, and results in an immunosuppressive phenotype (105). The single-cell RNA sequencing results from patients with GBM have revealed the broad expression of PD-L1 on TAMs, indicating that PD-L1 represent a potential target in GBM treatment (106, 107). A recent randomized phase III study (NCT02017717) was the first of its kind to investigate the therapeutic effect of nivolumab (a PD-1 inhibitor) in recurrent GBM (108). Unfortunately, this strategy did not result in a better outcome than treatment with bevacizumab. Similarly, nivolumab failed to benefit patients with resectable GBM in a phase II clinical trial (NCT02550249) (109). However, preoperative treatment with the neoadjuvant pembrolizumab (a PD-1 inhibitor) induced IFN-γ expression and significantly extended overall survival in another study of recurrent GBM (110). In addition, many clinical trials combining PD-1 inhibitors with other agents are underway, such as the CSF-1/CSF-1R inhibitor (NCT02526017), CTLA-4 inhibitor (NCT03367715), IDO1 inhibitor (NCT04047706), TIM-3 inhibitor (NCT03961971).

Although several immune checkpoint inhibitors have been tested in clinical trials, the number of patients with GBM that benefit is still limited. Understanding the mechanism of potential drug resistance and identifying patients who are more likely to respond to this strategy are the main challenges facing the development of effective GBM treatment strategies.

## 4 TAM-targeting translational research in GBM

**Figure 3:**
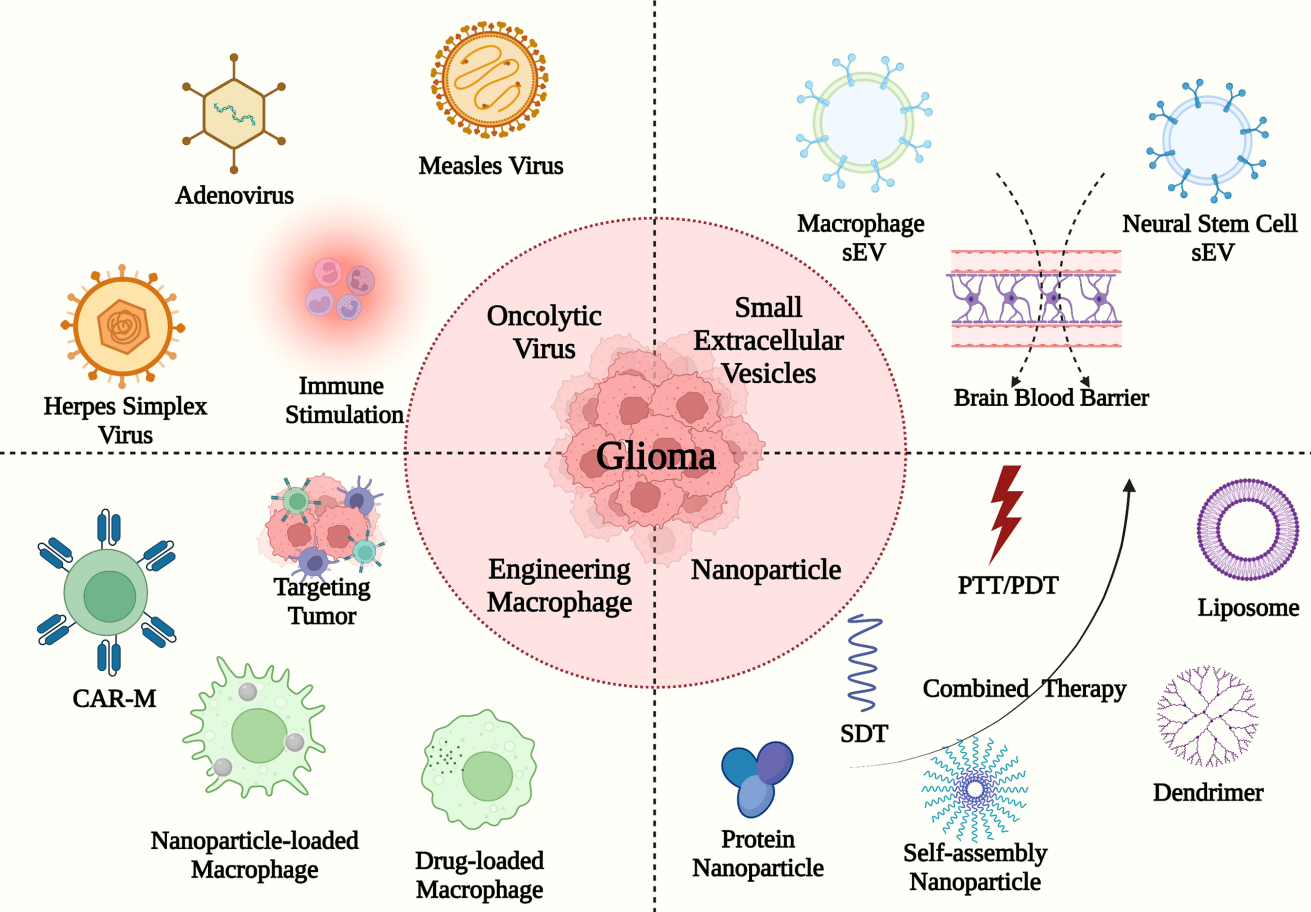
The TAM-targeting immune translational studies in GBM. The deliveries consist of oncolytic virus (e.g., herpes simplex virus, measles virus, and adenovirus), sEVs (e.g., macrophage sEV and neural stem cell sEV), engineering macrophages and nanoparticles with PTT/PDT/SDT (e.g., liposome, dendrimer, protein nanoparticle, and self-assembly nanoparticle).

Although several approaches targeting TAMs in GBM have been applied to enhance antitumor immune cell responses in clinical experiments, the patient prognosis remains unsatisfactory. In this section, we review the findings of translational studies documenting the development of novel bioactive agents such as the oncolytic virus, the small extracellular vesicles (sEV), and nanoparticles (NPs) for the treatment of GBM (**Figure 3** and **Table 2**).

**Table 2:** TAM-targeting translational researches in GBM.

Delivery types	Delivery advantages	Active components	Delivery platforms	Assisted treatments	Immunological effects	References
**Oncolytic Virus**	GBM targeting, Immune stimulation, BBB permeability	HSV	—	Trametinib	TAMs reduction	(111)
		ICOVIR17	—	Anti-PD-1	M1 polarization, TME regulation, ECM degeneration, reduction in immune escape	(112)
		Delta24-RGD	—	—	M1 polarization, TME regulation	(113)
		NAP	MV	Ruxolitinib Anti-PD-1	M1 polarization, TME regulation, reduction in immune escape	(41)
**Small Extracellular Vesicle**	Long circulating half-life, BBB permeability, high biocompatibility	CPPO, Ce6, AQ4N	Macrophage sEV	—	M1 polarization	(114)
		DOX	Macrophage sEV	—	TME regulation	(115)
		CAT, ICG	Macrophage sEV	SDT	—	(116)
		STAT3ASO, CpG	NSCs sEV	—	M1 polarization, reduction in immune escape	(117)
**Engineering Macrophage**	GBM targeting, BBB permeability, high biocompatibility	DOX	Engineering monocyte	—	M1 polarization	(118)
		rAAV2-IL-15	Engineering MG	—	NK cells activation	(119)
		Fe_3_O_4_	Engineering BMDMs	PTT	—	(120)
		TMZ	Engineering BMDMs	—	—	(121)
		—	CAR-M	—	M1 polarization	(122)
**Nanoparticle**						
**Organic NPs**	Biocompatibility, stability	miR155	Erythrocyte membrane coated NPs	—	M1 polarization	(123)
		IR-792	Macrophage membrane coated NPs	PTT	—	(124)
		Honokiol, Disulfiram, Copper	Liposome	—	M1 polarization, TME regulation	(125)
		DOX, pDNA-CD47	Liposome	BNCT	TME regulation, reduction in immune escape	(126)
		Rg3, PTX	Liposome	—	M1 polarization, TME regulation	(127)
		shRNA-CD47	PAMAM	TMZ	M1 polarization	(128)
		Rapamycin	PAMAM	—	TME regulation	(129)
		Triptolide	PAMAM	—	TME regulation	(130)
		siRNA-STAT3	SPNP	IR	M1 polarization, TME regulation	(131)
		Disulfiram, Cu, Regorafenib	T12/Man-BSA NPs	—	M1 polarization, TME regulation	(132)
		Simvastatin, Fenretinide	T/LF NPs	—	M1 polarization, TME regulation	(133)
		PTX, CpG	PNPPTX/MNPCpG	—	M1 polarization, TME regulation	(134)
		CpG, Plerixafor	CpG NPs/AMD-Zn	—	TAMs reduction, TME regulation	(135)
		PTX, R837	TfR/TATH7/PTX/R837 NMs	—	M1 polarization, TME regulation	(136)
		IRF5 mRNA, IKKβ mRNA	IRF5/IKKβ-encoding NPs	—	M1 polarization	(137)
		Anti-PD-1, Anti-CTLA-4	P/a-PD-1, P/a-CTLA-4	—	M1 polarization, reduction in immune escape, TME regulation	(138)
		Anti-PD-L1	Gluc-S-aPD-L1	—	M1 polarization, reduction in immune escape	(139)
		Ce6, Anti-PD-L1	Ce6-αPD-L1	PDT	M1 polarization, reduction in immune escape, TME regulation	(140)
**Inorganic NPs**	Controlled size and shape, surface plasticity	CpG	Au NPs	Anti-PD-1, RT	M1 polarization, reduction in immune escape, TME regulation	(141)
**Organic/inorganic Nanohybrids**	Biocompatibility, stability, controlled size and shape, surface plasticity	MnO2, Pt	MPM@P NGs	—	—	(142)

Novel noninvasive physical approaches with few toxic adverse effects have been used to eliminate GBM cells in translational studies, including photothermal therapy(PTT) (120, 124), photodynamic therapy(PDT) (140), and sonodynamic therapy(SDT) (116). Phototherapy consisting of PTT and PDT, relies on phototherapeutic agents and light irradiation to kill tumor cells in the dark (143). PTT involves the heat generated by photothermal agents under the appropriate near-infrared light (144). In turn, the photosensitizers under the appropriate light boost the reactive oxygen species (ROS) production in tumor cell, which is an important anti-tumor mechanism of PDT (145). Moreover, SDT is a potential approach with high tissue-penetrating properties based on ultrasound and sonosensitizers. Similar to PDT, the ROS-based effect is one of the action mechanisms in SDT. Besides, cell membrane disruption and lipid peroxidation are the main reasons for cell death. However, the underlying mechanisms of SDT hinder the process of clinical translation, which merits further investigation.

### 4.1 Oncolytic virus

As an emerging cancer immunotherapy, the oncolytic virus can selectively replicate in tumor cells while avoiding harming healthy tissues. Oncolytic viruses bind to tumor surface receptors and trigger immunogenic cell death (ICD), leading to the release of DAMPs, pathogen-associated molecular patterns (PAMPs), and tumor-associated antigens (TAAs). This in turn switches the TAM phenotype from M2 to M1 (146, 147). In 2015, the FDA approved the first oncolytic virus-mediated immune therapy, talimogene laherparepvec (T-VEC), for use in a phase III randomized controlled trial of phase III−IV melanoma (NCT00769704). In this study, T-VEC was engineered using herpes simplex virus type 1 (HSV-1), which promoted the release of granulocyte-macrophage colony-stimulating factor (GM-CSF) (148). In recent years, TAM-targeting oncolytic viral immunotherapies have been investigated for the treatment of GBM. The activation of MEK-ERK signaling was shown to promote the progression of malignant tumors, and HSV-1 increased the transport of the MEK1/2 kinase inhibitor, trametinib, across the BBB, leading to a reduction in the total number of TAMs at the tumor site. The consequent activation of CD8^+^ T cell-mediated immunity promoted the survival of GL261 model mice (111).

Hyaluronan (HA) is considered the main TME-contributing component of the extracellular matrix (ECM). The oncolytic adenovirus ICOVIR17 combined with anti-PD-1 antibodies could degrade HA, activate the NF-kB signaling pathway, triple the number of M1 macrophages, and prolong the median survival time of GL261 model mice to 43.5 days (112). Additionally, van den Bossche et al. reported the application of oncolytic virotherapy (Delta24-RGD) for improving the local immune microenvironment in GBM by promoting the secretion of TNF-α, IL-6, and IL-8 (113). Moreover, ruxolitinib directly inhibited the Janus kinase 1/2 (JAK1/2) and consequently blocked the JAK/STAT signaling pathway, thus lowering the expression of pro-proliferative, antiapoptotic, and immunosuppressive proteins and overcoming the resistance to viral replication in GBM. An oncolytic measles virus (MV) platform was modified with the aid of the *H. pylori*-derived NAP to activate macrophages in GBM *via* TLR2 targeting and promoting the secretion of the high-mobility group box1 protein (HMGB1). Furthermore, the combination of ruxolitinib, MV, and anti-PD-1 antibodies markedly enhanced the adaptive immune response and prevented tumor cells from orchestrating immune escape (41). Despite the promising outcome, this form of treatment has some inevitable drawbacks: the long-term inhibition of IFN secretion caused by ruxolitinib might generate an immunosuppressive microenvironment and the intra-tumoral injection of an oncolytic virus could serve as an obstacle to the clinical application of this strategy in GBM. Despite these drawbacks, the therapeutic applications of oncolytic viruses in GBM immunotherapy have bright prospects due to the unique tumor targeting properties of these viruses, which help drugs cross the BBB and induce local immune responses. Therefore, clinical translational studies should endeavor to construct modified oncolytic viruses to overcome the limitations of conventional drug delivery systems.

### 4.2 Sev

sEVs originate from intraluminal vesicles (ILVs) that are secreted by intracellular multivesicular bodies (MVBs). These extracellular organelles have a diameter of 40−160nm and are characterized by a long circulating half-life, efficient BBB penetration capability, and high biocompatibility (149). In clinical trials, sEVs containing RNA, DNA, and protein, have been considered biomarkers to diagnose non-small cell lung cancers (NSCLCs), prostate cancers, and other diseases. In addition, loaded sEVs have been used as drug delivery vectors in the treatment of pancreatic cancer (149–151). Drug-loaded M1 macrophage-derived sEVs have recently been shown to switch the phenotype of TAMs from M2 to M1 in GBM by promoting the secretion of TNF-α, IL-6, IFN-γ, and IL-1β from GBM cells. The 2,4,5-trichloro-6-carbopentoxyphenyl oxalate (CPPO), chlorin e6 (Ce6), and banoxantrone (AQ4N) drugs were delivered encapsulated in M1 macrophage-derived sEVs as a form of chemiexcited photodynamic therapy (CDT). This sEV-based treatment approach extended the median survival of GBM model mice to 40 days (114). Because focused ultrasound (FUS) can reversibly and transiently disrupt the BBB to help sEVs access the cranial cavity, Bai et al. developed a method whereby doxorubicin (DOX)-loaded macrophage-derived sEVs are combined with FUS to trigger ICD in mice with GBM (115). In another study, the performance of engineered macrophage-derived sEVs containing glutathione (GSH)-responsive silica nanoparticles (NPs) was evaluated in GBM mouse model. The silica NPs were coated with indocyanine green (ICG) and encapsulated with catalase (CAT), which led to oxygen generation and reversed the hypoxic TME of GBM (116). On a mechanistic level, ICG plays the role of a sonosensitizer in sonodynamic therapy (SDT) following ultrasound irradiation to increase the level of ROS, thus causing GBM cell necrosis. Meanwhile, CAT addressed the resistance of cancer cells to SDT, which is limited by hypoxia. In combination, ICG and ROS successfully enhanced SDT efficiency.

Neural stem cell (NSC)-derived sEVs were reported in TAM-targeting immunotherapies in GBM. The NSC-derived sEVs containing CpG-STAT3 antisense oligonucleotide (ASO) conjugates were delivered into GBM-resident macrophages, leading to M1 macrophage polarization. In addition, CpG acted as a TLR9 agonist to activate the NF-kB signaling pathway and increase IL-12 levels while lowering STAT3 expression, which together relieved the immunosuppressive effect on macrophages in the TME (117). In 2019, the FDA reported that several patients experienced serious adverse events after being treated with unapproved sEVs in the U.S. state of Nebraska, indicating that the clinical safety of sEVs needs to be improved (152). Moreover, despite their high degree of biocompatibility, sEVs have limitations associated with their capability to targeting tumor cells. Thus, future research should further explore the reasons for the adverse effects associated with exosomal delivery systems and optimize their capability to target GBM cells.

### 4.3 Engineering macrophages

Recently, exogenous engineering macrophages delivery has emerged as promising cancer immunotherapy (153). Furthermore, engineering monocytes, MG, and BMDMs have been applied for GBM therapeutic researches.

Monocytes can be preferably recruited into tumor site, cross the BBB, and target tumor, serving themselves as an ideal drug carrier with a long circulating half-life (153). An engineering monocyte containing functionalized nanodiamonds bearing DOX was delivered into GBM cells. The massive recruitment of monocytes helped DOX internalized by GBM cells and promoted the secretion of calreticulin (CRT), HMGB1, and ATP, inducing ICD and repolarizing resident M2 macrophages to M1 macrophages (118).

Similarly, BMDMs and MG can directly cross the BBB to target GBM cells by binding to the vascular cell adhesion molecule-1 (VCAM-1). The engineering MG transfected with recombinant adeno-associated virus serotype 2 containing IL-15 were intranasally administrated to promote the maturation and survival of NK cells by IL-15 to kill tumor cells (119). Additionally, Wang et al. developed an engineering BMDM carried with a photothermal nanoprobe Fe_3_O_4_ for postoperative photothermal therapy (PTT) in rats. Fe_3_O_4_ – loaded BMDM were injected into the tail vein and reached the surrounding tumor achieving PTT under 808 nm near-infrared (NIR) light irradiation (120). Recently, a noninvasive gut-to-brain oral prodrug BMDMs delivery system was developed for GBM treatment. The prodrug self-assembly nanoparticles conjugating TMZ with β-glucans could bind with the phagocytic pattern-recognition receptor Dectin-1 which was expressed on intestinal microfold cells and macrophages. Therefore, this self-assemble nanoparticle overcame the limitation of the intestinal epithelial barrier (IEB) and BBB and released TMZ in tumor sites (121). However, low drug loading efficiency and unstable drug release limited the application of engineering macrophages as drug delivery.

In addition, chimeric antigen receptors macrophages (CAR-M) were shown as an efficient immunotherapy for GBM. Chen et al. reported a method to engineer GSCs-CAR-M in the postsurgical cavity (122). The pentaspan transmembrane glycoprotein CD133, a marker for GSCs, was related to tumor progression, metastasis, and recurrence (154). They constructed a nanoporter-hydrogel to induce the local MG and BMDMs into CD133-CAR-M, resulting in recognizing and eradicating GSCs. Compared to CAR-T cells, CAR-M normally have difficulties in expanding *in vitro*, which hinders the process of clinical translation. In contrast, this novel strategy seems to be a guideline for CAR-M construction in GBM treatment.

### 4.4 Nanoparticles

Based on the components of nanoparticles, NPs classically are divided into inorganic NPs, organic NPs, and organic/inorganic nanohybrids. Here, we discuss different types of NPs applications in TAM-targeting treatment for GBM.

#### 4.4.1 Organic NPs

Biocompatible organic NPs are ideal carriers for drug and gene encapsulation (155, 156). In GBM, various organic NPs such as liposomes, dendrimers, and protein nanoparticles have been used in targeting TAM immunotherapies.

NPs are delivered into the tumor by the enhanced permeability and retention (EPR) effect, while they are normally recognized as foreign bodies in the liver, spleen, and kidneys. Organic, cell-membrane-coated NPs have advantages such as immune evasion, active targeting, and high biocompatibility (157). Gao et al. reported a virus-mimicking erythrocyte-membrane-coated nanogel containing the microRNA miR155 and targeting macrophages and MG *via* the inclusion of M2pep and HA2 peptides. In GBM, miR155 has been shown to promote the secretion of pro-inflammatory cytokines such as IL-6 and TNF-α, the overexpression of iNOS (a M1 phenotype marker), and the reduction in CD206 (a M2 phenotype marker) levels (123). The flow cytometric analysis showed that the number of M1 macrophages in the viral-mimic nanogel group was about four times higher than that in the control group, while the median survival time was prolonged to 27.6 days in the group of mice receiving the viral-mimic nanogel (123). In addition, another study evaluated the capacity of macrophage-membrane-coated NPs loaded with IR-792 to induce PTT in mice with GBM under NIR-Ib (900−1000 nm) light irradiation (124). However, methods that involve producing NPs using the cell membranes are still under development, and the disruption of the NP cell membrane integrity results in different endocytic mechanisms. The current research identified that the high coating degree (≥ 50%) NPs were internalized individually and the low coating degree (< 50%) NPs needed to be aggregated together to enter cancer cells (158). Therefore, cell membrane coating technologies need to be optimized in terms of mass production efficiency and quality before consistent results can be achieved in a clinical setting.

The liposome is one of the most promising nano-carriers in cancer therapy; it is normally used to deliver RNA because of its positive charge. The first FDA-approved siRNA lipid NP drug (Onpattro) has been used in clinical practice (159). A liposomal disulfiram/copper complex and honokiol co-delivery system have been developed with the aim of remodeling the immune TME of GBM. Honokiol promoted the release of IFN-γ *via* inhibiting the PI3K/mTOR pathway, while the disulfiram/copper complex increased the expression levels of CRT, ATP, and TNF-α in the TME. As a result, IL-6 secretion was reduced while the number of M1 macrophages doubled (125). Moreover, a multifunctional liposome delivery system, encapsulating DOX and carborane, was reported to enhance the immunotherapy of GBM (126). CD47, a receptor overexpressed on tumor cells, recognized the signal-regulatory protein α (SIRPα) on macrophages and protected the tumor cells from phagocytosis (160, 161). The immunosuppressive TME was shaped by antigen presentation due to DOX-induced ICD, and the reduction in CD47 and the boron neutron capture therapy (BNCT) enhanced immune surveillance (126). Additionally, Zhu et al. constructed a ginsenoside Rg3 (Rg3)-based liposomal system containing paclitaxel (PTX) to remodel the immunosuppressive TME of GBM. The combined treatment promoted the polarization of macrophages towards the M1 phenotype, decreased the number of M2 macrophages (to one quarter of those in the control group), and significantly reduced the number of regulatory T cells (Tregs) (127). Recently, selectively-targeting liposomes were constructed that were able to accurately deliver their contents to a specific organ such as the liver, lung, and spleen (162). Therefore, a brain-targeting liposome delivery system could be a potential strategy for the treatment of GBM.

Dendrimers are regarded as promising drug-delivery NPs in medical applications due to their stable structure and multivalent cooperativity. Poly(amidoamine) or PAMAM is one of the most intensively studied dendrimers in current biomedical applications (163). Song et al. reported the use of an implantable PAMAM-containing hydrogel loaded with shRNA in a mouse model of postoperative GBM. GBM cells reduced the expression of CD47 after being exposed to shCD47-loaded PAMAM, which made them more suspectable to immunosurveillance. The application of PAMAM-containing hydrogel to the tumor converted M2 to M1 macrophages and prolonged the median survival of model mice treated with TMZ to 73.0 days (128). Notably, a TAM-targeting PAMAM loaded with rapamycin could lead to a reduction in *Arg1* expression and immunosuppressive TME remodeling (129). Another study documented the application of the TAM-targeting PAMAM-containing triptolide to reshape the GBM TME *via* the inhibition of STAT3 activity (130). The main problem of the PAMAM delivery system is biotoxicity, which has delayed its clinical application.

Human serum albumin (HSA) was used to construct GBM-targeting protein NPs and inhibit tumor proliferation and invasion *via* the delivery of siSTAT3 (131). In this study, the number of M1 macrophages in the experimental group (combined with radiotherapy) increased to 2.5 times of that in the control group. Moreover, dual-targeting biomimetic albumin NPs encapsulated with regorafenib, and disulfiram/copper complex had been developed to remodel the immunosuppressive TME. In this setup, the albumin NPs can target the GBM cells and tumor vessel endothelial cells to regulate the TME, while the disulfiram/copper complex induced ICD to promote antigen presentation and regorafenib repolarized the M2 macrophages to assume the M1 phenotype (132). Mo et al. used lactoferrin biomimetic nanoparticles to send simvastatin and fenretinide into the GBM (133). Here, fenretinide directly generated excess ROS to promote tumor apoptosis, suppressed the function of M2 TAMs, and inhibited angiogenesis. Meanwhile, simvastatin cooperated with fenretinide to prevent GBM growth and increase the proportion of M1 macrophages in the TME, thus prolonging the median survival to 45 days. The NPs containing human proteins display a high degree of biocompatibility and specificity and therefore represent promising tools for the treatment of GBM.

NPs simultaneously targeting GBM cells and macrophages prolonged the median survival of mice to 50 days. When glutathione (GSH)-responsive PTX prodrug nanoparticles were delivered into GBM cells, the PTX induced ICD *via* the secretion of HMGB1, which abrogated the TME-mediated immunosuppression in GBM. Besides, the mannose-modified immunoadjuvant CpG NPs directly targeted macrophages to increase the number of M1 TAMs and reduce the proportion of Tregs within the TME, while also boosting IL-12, TNF-α, and IFN-γ expression (134). Zhang et al. reported an injectable hydrogel containing two different kinds of NPs in postoperative GBM treatment (135). The CpG oligonucleotide NPs activated the polarization of M1 TAMs *via* the activation of TLR9 to regulate the local microenvironment, while the self-assembly NPs containing plerixafor downregulated the expression of CXCR4 and reduced the secretion of CXCL12 leading to the inhibition of M2 TAM recruitment. Furthermore, the use of two kinds of pH-sensitive self-assembling nano-composite micelles encapsulated with PTX and imiquimod (R837) in localized GBM therapy prolonged the median survival time of mice to 66 days (136). The R837 resulted in M2 to M1 macrophage polarization and promoted the secretion of cytokines like TNF-α. Additionally, Zhang et al. constructed the TAM-targeted mRNA self-assembling NPs by exploiting the electrostatic interactions between the anionic mRNA and the cationic polymer. These NPs reprogrammed macrophages in GBM by lowering the expression of interferon regulatory factor 5 (IRF5) and IkappaB kinase beta (IKKβ) (137). IRF5 directly boosted inflammatory cytokines expression, such as IL-12, IL-6, IL-23, and IFN-γ, while IKKβ regulated the inflammatory TME through the activation of NF-κB (164, 165). The components of self-assembling NPs normally play different roles in tumor treatment while certain NPs synthesis methods may contain toxic components that trigger inevitable adverse effects. Therefore, the self-assembly strategy is a promising approach for building biocompatible NP-associated drug and gene delivery systems.

BBB permeable nanoscale immunoconjugates were invented to increase the concentration of therapeutic antibodies in the brain (138). The angiopep-2 (AP-2) peptide conjugated to poly (β-L-malic acid; PMLA) and the capacity to cross the BBB *via* transferrin receptor (TfR)-mediated transcytosis (138). Meanwhile, the combination of anti-CTLA-4 and anti-PD-1 antibodies relieved the immunosuppression of the GBM TME by promoting the secretion of cytokines like IL-1β, IL-2, IL-12, and TNF-α, which increased the proportion of M1 polarized TAMs and enhanced the immunosurveillance at the tumor site. Similarly, Yang et al. added multiple polyethylene glycol (PEG) chains to anti-PD-L1 antibodies to enhance the potency of conventional GBM-targeting ICB therapy *via* the overexpression of glucose transporter-1 on the GBM vasculature (139). Furthermore, the IFN-γ levels in the engineered Gluc-S-aPD-L1 group were ~1.9−3.0 times higher than in the control group. In another study, the self-assembling nanocomplexes (~30 nm in diameter) were loaded with Ce6 and anti-PD-L1 antibodies and extended the median survival of mice to 32 days (140). The photosensitizer Ce6 induced photodynamic therapy (PDT) under 660 nm light irradiation and presented antigens to immune cells, while the combined treatment with anti-PD-L1 antibodies increased the M1/M2 ratio to 1.88 and reduced the proportion of immunosuppressive cells like Tregs. Tumor-targeting immunoconjugates composed of antibodies with the permeability across the BBB overcome the limitation of traditional ICB therapy; thus, their clinical value should be investigated after addressing the challenges encountered during production.

#### 4.4.2 Inorganic NPs

The size, shape, and surface characteristics of inorganic NPs can be tightly regulated, endowing these vesicles with useful properties for use in clinical imaging, diagnosis, and treatment (155, 156). Gold NPs are the most common type of inorganic NP used for drug delivery. Cao et al. described the efficacy of TAM-targeting CpG-modified gold NPs for use in radio-immunotherapy (141). In this study, the TLR9 agonist CpG promoted the overexpression of iNOS and IL-12, while the combined treatment with anti-PD-L1 antibodies reeducated macrophages in GBM and enhanced the response to radiotherapy. The diameter of gold NPs determines their high rates of renal clearance and hepatotoxicity which prevents these NPs from reaching further clinical efficacy. Thus, the application of inorganic NPs in GBM treatment is promising but requires optimization.

#### 4.4.3 Organic/inorganic nanohybrids

The organic/inorganic nanohybrids combine the typical properties of organic and inorganic NPs to produce NPs with new properties (156). For example, Xiao et al. designed the macrophage-membrane-coated MnO_2_ NPs carrying cisplatin (Pt) to induce chemodynamic therapy (CDT). In this design, the macrophage membrane acts as the organic component and permits BBB penetration and GBM targeting, while the inorganic material MnO_2_ triggers the Fenton-like reaction to enhance the efficacy of CDT (142). Although studies documenting the application of nanohybrid in GBM treatment are scarce, the combination of organic and inorganic materials will generate novel effects that could improve GBM outcomes in future.

## 5 Discussion and outlook

The simultaneous existence of BMDMs and MG highlights the complex interactions between GBM and TAMs. Therefore, the roles of BMDMs and MG in GBM proliferation, metastasis, and angiogenesis, and especially the relationship between resident MG and GBM cells, need to be further explored. Moreover, the development of the immunosuppressive TME, to which TAMs contribute, represents a major obstacle to the implementation of GBM-targeting immunotherapies

The TAM-targeting therapies evaluated in clinical trials can be divided into three strategies: those that target i) TAM recruitment; ii) TAM polarization, or iii) immune checkpoints. CSF1R inhibitors, the quintessential TAM recruitment inhibitors, cannot prolong patients’ survival time alone, although they may be useful in combination with other treatment forms. As we have seen, certain ANG2 inhibitors and CXCR4 inhibitors exert favorable effects on preventing GBM growth. In addition, the TLR agonists markedly improved the prognosis of patients with GBM by switching TAMs from the M2 to the M1 phenotype. Furthermore, TLR agonists are also being used in tumor vaccines as immune adjuvants. However, ICB including the use of anti-PD-1 and anti-PD-L1 antibodies, whose limitations and challenges still need to be illustrated, have for various reasons failed in GBM clinical trials. Thus, the future potential impact of TAM-targeting strategies should not be underestimated.

To overcome the pitfalls of existing clinical immunotherapies, numerous translational studies are concentrating on targeting TAMs in GBM. These include the oncolytic virus, sEVs, engineered macrophages, and NPs. In recent years, both the oncolytic viral vectors and sEVs have become promising clinical translational candidates However, engineering macrophages with BBB permeability is limited by the current macrophage extraction technology and the current inevitable adverse effects; future solutions will have a significant impact on clinical translation. NPs normally are characterized by a high degree of biocompatibility, stability, surface plasticity, and ease of availability. Although the therapeutic effects of NPs have been verified in animal models, their use in clinical trials typically presents a challenge.

In conclusion, the rapid development of TAM-targeting immunotherapy will revolutionize the standard care of patients with GBM in the future. Clinicians should therefore fully appreciate the limitations of this promising strategy in clinical practice while harnessing its value for the treatment of GBM.

## Author contributions

ZW, HZ, XL, and SN wrote the manuscript and provided final approval for the version to be published. XL and SN provided the supervision of the entire work. All authors contributed to the article and approved the submitted version.

## Funding

This work was supported by National Natural Science Foundation of China (82172740, 82111530202, 81874082), the Shandong Provincial Natural Science Foundation (ZR2021LSW008, ZR2020QH234), the Department of Science & Technology of Shandong Province (ZR2019ZD33, 2020CXGC010903), the Special Foundation for Taishan Scholars (ts20110814, tspd20210322), the Innovation Project of Jinan Science and Technology Bureau (2021GXRC065), Clinical Research Center of Shandong University (2020SDUCRCB002), Research Project of Jinan Microecological Biomedicine Shandong Laboratory (JNL-2022003A and JNL-2022042C), and the Rongxiang Regenerative Medical Fund (2019SDRX-19), Shandong University multidisciplinary research and innovation team of young scholars (2020QNQT001).

## Acknowledgments

We would like to thank BioRender for all figures created with BioRender.com.

## Conflict of interest

The authors declare that the research was conducted in the absence of any commercial or financial relationships that could be construed as a potential conflict of interest.

## Publisher’s note

All claims expressed in this article are solely those of the authors and do not necessarily represent those of their affiliated organizations, or those of the publisher, the editors and the reviewers. Any product that may be evaluated in this article, or claim that may be made by its manufacturer, is not guaranteed or endorsed by the publisher.
